# Immune regulation and therapeutic targets in sepsis: insights from single-cell transcriptomics

**DOI:** 10.3389/fimmu.2026.1839417

**Published:** 2026-05-28

**Authors:** Ruijuan Zhang, Xueping Lou, Huihui Huang, Jiaxu Zhou, Qi Dai, Zhencang Zheng

**Affiliations:** 1 Department of Critical Care Medicine, Taizhou Central Hospital (Taizhou University Hospital), Taizhou, Zhejiang, China; 2 Taizhou Hospital of Zhejiang Province, Taizhou, Zhejiang, China; 3 College of Life Sciences, Zhejiang Sci-Tech University, Hangzhou, China

**Keywords:** biomarkers, immune regulation, sepsis, single-cell transcriptomics, therapeutic targets

## Abstract

Sepsis is a life-threatening systemic inflammatory syndrome often associated with immune dysregulation, multi-organ dysfunction, and high mortality. In recent years, single-cell transcriptomics has generated a wealth of data on immune cell heterogeneity, lineage reprogramming, and functional states in sepsis. However, a systematic integration and summary of immune cell subsets and regulatory mechanisms are still lacking, limiting a comprehensive understanding of dynamic immune responses and potential therapeutic targets across disease progression. This review synthesizes advances from single-cell and multi-omics studies in sepsis, highlighting myeloid-biased hematopoietic stem/progenitor cell differentiation and emergency granulopoiesis, immunosuppressive features of neutrophils and MDSCs, T/NK cell exhaustion and metabolic adaptation, B cell and plasma cell reprogramming, and glial cell polarization affecting the blood-brain barrier. Key molecular mechanisms, including TLR4 acetylation, STAT3-CEBPB axis, Fu-Hp/Mincle signaling, Treg metabolism, and the kynurenine pathway, are discussed, alongside potential biomarkers and therapeutic targets such as inflammatory CD16^+^ monocytes, SRS1 neutrophils, PICS-associated T/B cells, and polarized glial cells. This integrated perspective provides a framework for understanding systemic and central immune regulation in sepsis, underscores the importance of dynamic, multi-omics, and spatial analyses, and establishes a foundation for early diagnosis, prognosis evaluation, and precision immunotherapy.

## 1 Introduction

Sepsis is defined as life-threatening organ dysfunction caused by a dysregulated host response to infection and remains the leading cause of death among critically ill patients (1). Despite advances in supportive care, mortality from sepsis remains unacceptably high, with fatality rates reaching up to 40% in patients with septic shock (2, 3). Long-term outcomes are equally concerning: one-year mortality among sepsis survivors remains significantly elevated, and risks of hospital readmission as well as cardiovascular and cerebrovascular complications are markedly increased (4, 5). Over the past decades, supportive treatment strategies—including appropriate antibiotic therapy, fluid resuscitation, and organ support—have been progressively optimized. However, specific and effective targeted therapies for sepsis are still lacking (6, 7). Immune dysregulation is now recognized as a central driver of sepsis pathogenesis, involving both excessive inflammation and persistent immunosuppression (8). However, clinical trials targeting either hyperinflammation or immune suppression have yielded limited and inconsistent efficacy (9–11), highlighting the need for deeper mechanistic understanding of immune heterogeneity in sepsis.

Traditional transcriptomic studies have largely relied on bulk RNA sequencing. While these approaches revealed extensive changes in inflammation-related gene expression, they could not distinguish the contributions of distinct cell types, thereby limiting comprehensive characterization of immune cell heterogeneity and functional state transitions. The advent of single-cell RNA sequencing (scRNA-seq) has enabled high-resolution profiling of immune cell landscapes and dynamic state trajectories at the single-cell level, offering unprecedented insight into the immunoregulatory mechanisms underlying sepsis. Since the first commercial platform was introduced in 2014 (12), rapid technological advances have expanded throughput and reduced cost, resulting in widespread adoption of scRNA-seq and the development of multiple high-throughput platforms (13, 14). This progress has driven exponential growth in single-cell datasets and analytical tools, with nearly 2,000 studies and thousands of computational methods now available (15–18). Beyond methodological development, single-cell technologies have been widely applied to diverse biological contexts, including development, disease progression, immune regulation, and rare cell identification (19–25). Concurrently, the number of computational tools for scRNA-seq analysis has expanded dramatically and is projected to reach 3,000 by the end of 2025 (26).

In the context of sepsis, single-cell and multi-omics studies have revealed extensive immune cell remodeling across disease stages and tissues. These include persistent inflammatory and immunosuppressive programs in late-stage disease, coordinated dysfunction of myeloid and lymphoid compartments, and dynamic neutrophil expansion during acute infection (27–32).

Recent multi-omics and spatially informed studies further demonstrate tissue- and organ-specific immune regulation, metabolic reprogramming of T cells, and neuro-immune interactions involving the central nervous system (33–35). At the mechanistic level, post-translational and glycosylation modifications have been shown to regulate inflammatory signaling intensity, while concepts such as “extreme response endotype” have been proposed to capture systemic immune heterogeneity (36–38). In parallel, single-cell-derived molecular signatures have been increasingly applied for biomarker discovery and risk stratification in clinical subgroups, including acute respiratory distress syndrome (ARDS) and neonatal sepsis (39, 40). Collectively, these advances highlight the transition of sepsis research from bulk population-level profiling to integrated single-cell and multi-omics frameworks.

Therefore, this review systematically summarizes recent advances in single-cell and multi-omics studies of sepsis, focusing on immune cell heterogeneity, molecular regulatory networks, multi-dataset integration, and emerging biomarkers. By integrating current datasets and analytical frameworks, we aim to provide a conceptual foundation for understanding immune dysregulation in sepsis and to support future development of precision immunotherapeutic strategies.

## 2 Overview of immune dysregulation in sepsis and the emerging role of single-cell technologies

### 2.1 Existing single-cell datasets in sepsis

**Figure 1:**
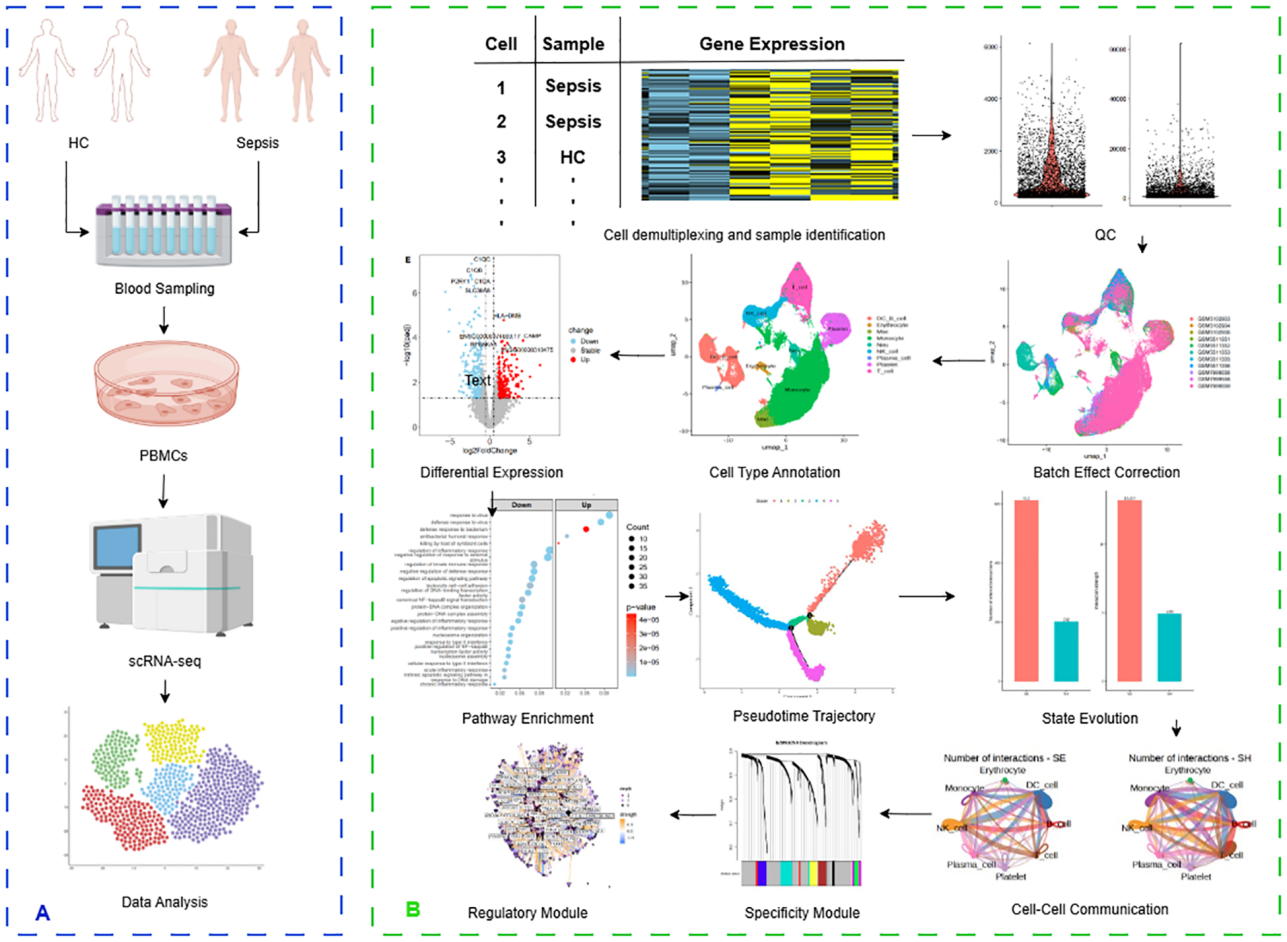
Schematic diagram of single-cell sequencing and analysis workflow for sepsis. **(A)** Workflow of sepsis sample collection, PBMC isolation, single-cell sequencing, and cell clustering analysis. **(B)** Single-cell sequencing data analysis workflow, including data preprocessing, cell type annotation, dynamic state analysis, cell–cell communication inference, and integrative model construction.

To systematically summarize currently available single-cell omics resources in sepsis immunology research, we conducted a comprehensive collection and curation of publicly accessible single-cell RNA sequencing (scRNA-seq) datasets up to 2025 (**Table 1**). Relevant datasets were primarily retrieved from public repositories, including the Gene Expression Omnibus (GEO), ArrayExpress, Single Cell Portal, Genome Sequence Archive/Human Genome Sequence Archive (GSA/Human GSA), and CNGBdb, along with integrative studies and multicenter clinical cohorts. Overall, these datasets predominantly focus on peripheral blood mononuclear cells (PBMCs), encompassing patients in the acute phase, recovery phase, and with varying degrees of disease severity (**Figure 1A**). Many datasets also include healthy controls, high-risk infected individuals who did not progress to sepsis, and selected tissue-derived samples. Collectively, these resources provide a critical foundation for dissecting immune heterogeneity and disease progression. Notably, cross-dataset comparisons reveal substantial variability in immune cell proportions and transcriptional signatures, largely attributable to differences in sequencing platforms, sample processing, disease stratification, and annotation strategies, highlighting the importance of integrative interpretation.

**Table 1:** Collected data of scRNA-seq including dataset, research focus, sample source, time point, and reference.

Dataset	Research	Sample source	Time point	Ref.
GSE242127	Hub Gene Signatures of Sepsis Coinfection with COVID-19 and Senile Sepsis-Induced ARDS	Peripheral blood from elderly patients with sepsis-induced ARDS, mouse CLP model, and public transcriptomic data of COVID-19 and sepsis	Single time point (acute phase) and dynamic comparison (Day 1 vs Day 7)	(39)
GSE236099	Immune signatures and diagnostic markers of preterm infant sepsis	Peripheral blood PBMCs and plasma from very preterm infants (<32 weeks)	Multiple time points (weekly and at infection events)	(40)°
GSE286921/GSE286922	Fucosylated Haptoglobin (Fu−Hp) Drives Sepsis Inflammation via the Mincle Receptor	Peripheral blood PBMCs and monocytes from sepsis patients and healthy controls	Single time point (acute phase)	(37)°
GSE175453	Non-myeloid immune cells in late-stage sepsis	Four sepsis patients (day 14–21) and five healthy controls	Single time point (late phase)	(27)
GSE167363	Dynamic changes of immune cells in early sepsis	Five patients with Gram-negative sepsis (0 h and 6 h) and healthy controls	Two time points (within 6 h after diagnosis)	(28)
GSE151263	Immune dynamics of sepsis secondary to bacterial pneumonia	Two patients (acute, stable, and recovery phases) and healthy controls	Multiple time points (full−course dynamic)	(29)
GSE252331	Myeloid cell responses following sepsis, particularly the heterogeneity of MDSCs	Healthy controls (n=12), acute sepsis (n=4), late sepsis (rapid recovery n=4, chronic critical illness CCI n=5)	Day 3–5, Day 14–21	(30)
GSE279452	Anatomical infection source and age-specific immune landscape	281 adult and pediatric sepsis patients and controls, multi-omics (scRNA-seq, CITE-seq, scTCR/BCR, proteomics)	Single time point (acute phase)	(33)
GSE290679	CD4+ T cell subset-specific metabolic adaptation and kynurenine metabolism	Peripheral blood from critically ill patients (sepsis and non-sepsis) and healthy adults	Mainly at 48–72 hours after admission	(34)
GSE307512	Single-nucleus transcriptomic landscape of sepsis-associated hippocampal neurovascular dysfunction	Post-mortem hippocampal tissues from sepsis and control patients	Single time point	(35)
Kwok et al., 2023	Neutrophils and emergency granulopoiesis drive immunosuppression and the SRS1 extreme response endotype	39 individuals (sepsis, healthy controls, sterile inflammation controls), whole blood and hematopoietic stem/progenitor cells (HSPCs)	Acute phase and recovery phase (1–6 months)	(38)
GSE268406	Role of TLR4-TIR domain acetylation in M1 macrophage polarization during sepsis	PBMCs from sepsis patients and mouse macrophages	Single time point (acute phase)	(36)
GSE217906	Immune cell characteristics of persistent inflammation, immunosuppression, and catabolism syndrome (PICS) after sepsis	Peripheral blood PBMCs from PICS patients, acute sepsis patients, and healthy controls	Single time point (PICS phase)	(31)
PRJCA039900	Single-cell transcriptomic landscape of peripheral blood immune cells during transition from high-risk state to clinical sepsis reveals early activation of monocytes and platelets	Peripheral blood PBMCs from healthy controls, high-risk individuals (e.g., those with fever due to urinary tract infection), and patients with clinical sepsis	Single time point (high-risk phase, clinical sepsis phase)	(32)

Early-phase datasets have been instrumental in capturing dynamic immune responses. GSE167363, one of the earliest and most widely used datasets, includes PBMC samples collected at 0 h and 6 h after sepsis recognition, together with healthy controls (27). This dataset reveals rapid activation of interferon (IFN)-related pathways in monocytes and early functional suppression in T cells (28), supporting the concept of inflammation–immune imbalance in early sepsis. Additional studies report abnormal neutrophil expansion with increased immature subsets during the acute phase (29), as well as marked activation of monocytes and platelets during the transition from high-risk infection to clinically defined sepsis (32). However, discrepancies in neutrophil and monocyte distributions across studies are frequently observed, reflecting differences in sequencing platforms, cell enrichment strategies, and annotation criteria for transcriptionally similar but functionally distinct populations. Overall, these findings consistently indicate early myeloid activation coupled with adaptive immune suppression, although the relative contribution of specific subsets varies across cohorts.

Beyond the acute stage, multiple datasets characterize disease progression and immunosuppressive phases. Late-stage analyses demonstrate reduced CD4^+^ and CD8^+^ T-cell numbers, decreased cytotoxic activity, and increased regulatory T cells, indicating aggravated adaptive immunosuppression (30). Longitudinal studies further reveal sustained activation of IFN signaling alongside progressive T-cell exhaustion, supporting a dual-abnormality model in which persistent inflammation and immunosuppression coexist rather than occur sequentially (33). Additional datasets, including GSE175453 and GSE151263, extend these observations to ARDS-associated sepsis and immune-mediated organ injury (27, 29), while also highlighting pronounced heterogeneity within myeloid populations, particularly among myeloid-derived suppressor cells (MDSCs) (30).

To address limitations in sample size and improve robustness, recent studies increasingly focus on integrating multiple datasets to construct large-scale immune atlases. For example, the SC2sepsis database integrates datasets such as GSE167363 and GSE175453, enabling automated cell-type annotation and cross-study comparison. The Trueblood cohort from the Broad Institute includes PBMC and bone marrow samples from sepsis patients and healthy controls, providing enhanced statistical power for comparing circulating immune responses with hematopoietic alterations (57). In addition, longitudinal analyses demonstrate substantial differences in immune trajectories between survivors and non-survivors, underscoring the importance of temporal dynamics beyond static profiling (33).

Recent advances have further extended single-cell studies toward multi-omics integration and cross-tissue analysis. Integrated approaches have revealed anatomically specific immune features and metabolic reprogramming in sepsis (33, 34), as well as neurovascular dysfunction associated with immune dysregulation (35). Emerging frameworks based on single-cell multi-omics temporal networks enable more effective integration of heterogeneous datasets and provide dynamic insights into disease progression (58). In parallel, studies of the brain immune microenvironment highlight the critical role of glial cells in neuroimmune signaling and central nervous system dysfunction (59). In addition, emergency granulopoiesis has been linked to the formation of extreme response endotypes, further extending the application of single-cell approaches to molecular disease stratification (38). Controlled-access datasets, such as PRJCA039900 and HRA000287, provide valuable stage-specific resources that support these integrative analyses.

Cross-species and integrative transcriptomic studies offer complementary perspectives on immune dysregulation. Murine single-cell datasets enable controlled investigation of early immune dynamics and reveal rapid remodeling of immune compartments during sepsis progression (32). However, murine responses are typically more synchronized and dominated by acute innate activation, whereas human sepsis exhibits greater heterogeneity and more prolonged immune dysregulation (33). Integrative analyses combining scRNA-seq with bulk RNA-seq datasets have identified key immune subsets, such as FCGR3A^+^ macrophages, and enabled the construction of prognostic models (60). This strategy enhances robustness and translational relevance by linking high-resolution discovery with large-scale validation.

Beyond dissociated single-cell analyses, spatial transcriptomics provides critical insights into how tissue architecture and microenvironmental context shape immune-cell behavior in sepsis. By preserving spatial information, these approaches enable the mapping of immune-cell localization, cellular neighborhoods, and ligand-receptor interactions within intact tissues (61, 62). In the context of sepsis, spatial and spatially informed transcriptomic studies have begun to reveal organ-specific patterns of immune-cell infiltration across the lung, brain, and bone marrow. Spatial analyses of inflammatory tissue injury demonstrate that myeloid cells preferentially accumulate in regions of damage, where they interact with endothelial and stromal cells to amplify local inflammatory responses (63). In the lung, spatial proximity between neutrophils and epithelial cells is associated with enhanced chemokine signaling and barrier disruption in sepsis-associated acute lung injury (64), whereas in the brain, microglial clustering around vascular structures correlates with blood–brain barrier dysfunction and neuroinflammation in sepsis-associated encephalopathy (65).

Importantly, spatial context refines the interpretation of cell-cell communication networks. Ligand–receptor interactions inferred from single-cell data can be validated and contextualized through spatial proximity, enabling more accurate identification of functional signaling axes in inflamed tissues (46, 62). Moreover, spatial heterogeneity reveals that immune responses are not uniformly distributed but instead organized into localized niches with distinct activation states, particularly under conditions of systemic inflammation and organ dysfunction (46, 63). The integration of spatial transcriptomics with complementary modalities, including CITE-seq, metabolomics, and epigenomics, will be essential for constructing a comprehensive view of tissue-specific immune regulation in sepsis. Such integrative approaches are expected to clarify how systemic immune dysregulation translates into organ-specific pathology and may provide new targets for spatially informed therapeutic interventions (46, 61).

### 2.2 Common analytical pipelines and tools

Single-cell omics studies in sepsis generally follow a standardized analytical workflow encompassing data preprocessing, cell type annotation, functional analysis, dynamic trajectory inference, cell-cell communication analysis, and integrative modeling (**Figure 1B**). These approaches collectively provide a framework for interpreting heterogeneous immune responses.

Data preprocessing begins with rigorous quality control to address noise, technical variability, and batch effects inherent in single-cell RNA-seq data. Common metrics include the number of detected genes, total transcript counts, and mitochondrial gene proportions. Following quality control, normalization and highly variable gene selection are performed (41–43). Widely used frameworks such as Seurat (44) and Scanpy (45) incorporate multiple batch correction strategies, facilitating integration across datasets generated from different platforms.

Cell type annotation is essential for downstream analysis but remains challenging due to dynamic immune states in sepsis that blur canonical marker boundaries. To improve accuracy, automated tools such as SingleR (46), CellTypist (47, 48), and reference atlas-based projection methods (49) are commonly combined with manual curation. Nevertheless, inconsistencies in annotation across studies remain a major source of variability, particularly for transitional or functionally plastic immune populations.

Functional characterization is typically performed through differential expression analysis followed by pathway enrichment (e.g., GO, KEGG, Reactome) (50–52). Integration with bulk RNA-seq datasets enables cross-validation and enhances biological interpretation, revealing coordinated dysregulation of inflammatory, immunoregulatory, and metabolic pathways in sepsis.

To capture dynamic processes, pseudotime trajectory analysis tools such as Monocle3 (53) and Slingshot (54) are widely used to reconstruct immune cell state transitions, particularly along activation-to-exhaustion continua. In parallel, cell-cell communication analysis methods, including CellPhoneDB (55) and NicheNet (56), characterize ligand-receptor interaction networks and provide insights into immune coordination and dysregulation.

Finally, integrative modeling approaches combining single-cell and bulk transcriptomic data with machine learning algorithms enable the construction of immune stratification frameworks and prognostic models. These strategies bridge cellular heterogeneity with clinical outcomes and align with emerging multi-omics approaches in sepsis research.

## 3 Transcriptomic and functional characteristics of hematopoietic and immune cell subsets in sepsis

**Figure 2:**
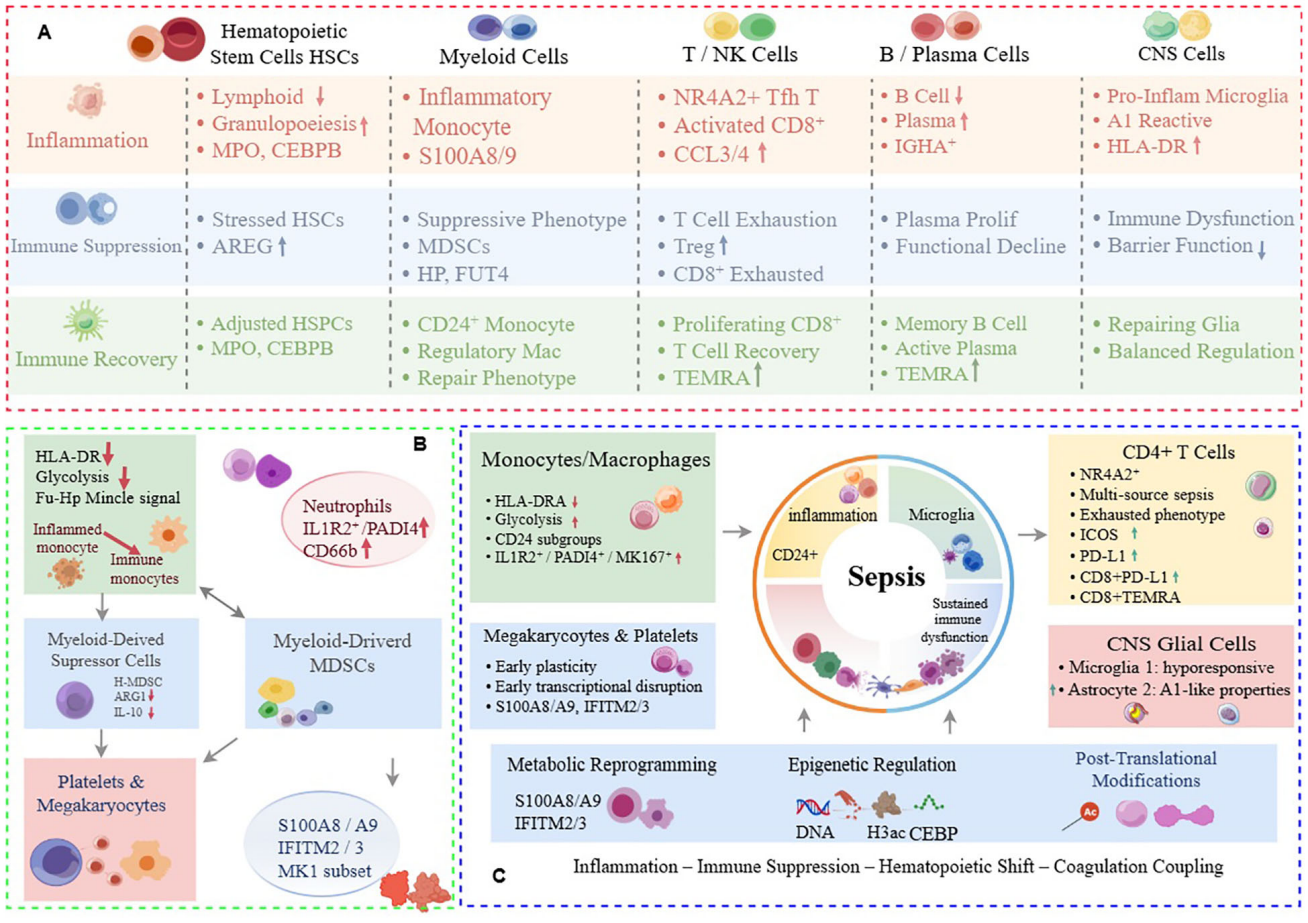
Analysis diagram of transcriptomic and functional characteristics of hematopoietic and immune cell subsets in sepsis. **(A)** Immune cell atlas of sepsis, highlighting the core information of single-cell subsets, disease stages, functional states, and key genes. **(B)** Myeloid cells form a dynamic axis of “inflammation–immune suppression–hematopoietic shift–coagulation coupling” in sepsis; monocytes/neutrophils/platelets/megakaryocytes act synergistically through metabolic reprogramming, transcriptional regulation, and post-translational modification to maintain sustained inflammatory activation and drive the chronic critical state. **(C)** Integrate the myeloid cell, lymphocyte glial cell, metabolism, epigenetics, PTM to construct a clear schematic diagram of the immune regulation mechanism in sepsis.

Sepsis induces extensive remodeling of the hematopoietic and immune systems, altering not only the composition of peripheral and tissue-resident cells but also their functional states. Single-cell multi-omics studies have shown that hematopoietic stem and progenitor cells (HSCs/HSPCs) display a bias toward myeloid differentiation, providing the basis for emergency granulopoiesis. Meanwhile, peripheral myeloid cells, including monocytes, macrophages, neutrophils, and myeloid-derived suppressor cells (MDSCs), undergo dynamic transcriptional reprogramming, transitioning from an early hyperinflammatory state to an immunosuppressive phenotype, thereby participating throughout the course of immune dysregulation in sepsis. Adaptive immune cells, such as CD4^+^ and CD8^+^ T cells, NK cells, as well as B cells and plasma cells, exhibit subset-specific exhaustion, metabolic remodeling, and functional impairment, further exacerbating systemic immune imbalance. In addition, glial cells in the central nervous system, including microglia and astrocytes, also display subset-specific functional alterations associated with neuroinflammation and blood-brain barrier disruption. Together, these changes reflect coordinated dysregulation across hematopoietic, immune, and neural compartments rather than isolated cell-type alterations (**Figure 2**).

### 3.1 Hematopoietic stem and progenitor cells

Single-cell multi-omics studies have revealed that sepsis not only reshapes peripheral immune cell composition but also profoundly influences the differentiation trajectories and functional states of hematopoietic stem and progenitor cells (HSCs/HSPCs) (30). In the circulation of patients with sepsis, the composition of HSCs is markedly altered, characterized by a reduction in lymphoid progenitors and an increased erythroid–myeloid differentiation bias, indicating an overall shift of the hematopoietic system toward myeloid responses. This “myeloid bias” is particularly evident in specific HSC subsets, such as the C5 and C7 clusters, which highly express granulopoiesis-related genes (e.g., MPO, RNASE2). Their chromatin accessibility profiles show strong enrichment for transcription factor motifs of the CEBP family (CEBPA/CEBPB) and STAT3, suggesting activation of enhanced granulopoietic programs, particularly emergency granulopoiesis (EG) (38). These observations indicate that systemic inflammatory signals associated with sepsis can propagate upstream to the hematopoietic hierarchy, reshaping HSC fate-determination networks and promoting granulocyte expansion and functional reprogramming at the source.

Notably, in scRNA-seq studies of neonatal sepsis in preterm infants, HSPCs and NK cells were identified as major sources of amphiregulin (AREG), which is significantly upregulated during the early phase of the disease (40). As a ligand of EGFR, AREG not only participates in tissue repair and barrier maintenance but may also influence the early balance between inflammation and tissue repair by regulating the hematopoietic microenvironment and intercellular immune communication. When considered together with the enhanced EG program observed in adult sepsis, these findings suggest that hematopoietic systems at different developmental stages adopt distinct adaptive strategies under inflammatory stress: adults primarily exhibit CEBP/STAT3-driven amplification of granulopoiesis, whereas preterm infants display developmental immune regulatory features mediated by AREG. These findings collectively emphasize that sepsis is a systemic disorder involving remodeling of the “hematopoiesis–immune axis,” with lineage reprogramming at the HSC level serving as a key mechanism that continuously supplies cellular sources for peripheral inflammation and immunosuppression.

### 3.2 Myeloid cells

Single-cell transcriptomic and multi-omics analyses consistently identify myeloid cells as central effectors of immune dysregulation in sepsis, with functional alterations spanning the entire disease course from early hyperinflammation to late-stage immunosuppression and chronic critical illness (**Figure 2B**). Across disease progression, these cells exhibit dynamic and coordinated transcriptional and functional reprogramming rather than discrete stage-specific behaviors.

In the early stage of sepsis, monocytes display a pronounced hyperinflammatory phenotype characterized by activation of inflammatory pathways and metabolic reprogramming. This state gradually transitions toward immunosuppression, marked by reduced HLA-DR expression and enhanced glycolytic activity (28, 29). During later or recovery phases, the emergence of subsets such as CD24^+^ monocytes suggests partial restoration of immune homeostasis and the initiation of immune reconstruction (27). Notably, pediatric pulmonary sepsis exhibits a distinct proliferative CD14^+^ monocyte subset (Mono_c06-CD14-MKI67) with high expression of MKI67, MPO, and ELANE, indicating a potential age-specific emergency monocyte response program (33). At the molecular level, post-translational modifications further shape monocyte/macrophage activation. Fucosylation of haptoglobin enhances signaling through the Mincle (CLEC4E) receptor, promoting differentiation into hyperinflammatory macrophage-like cells associated with poor prognosis (37), while CBP-mediated acetylation of the TLR4-TIR domain facilitates downstream inflammatory signaling and M1 polarization, particularly in CD16^+^ monocytes (36).

During disease progression, monocytes undergo continuous transcriptional reprogramming characterized by stage-dependent gene expression patterns. When high-risk conditions such as febrile pyelonephritis progress to sepsis, genes including S100A8, S100A9, IFITM2, and IFITM3 become persistently upregulated (32). Trajectory analyses suggest sequential activation programs, with S100A8/A9 peaking during early differentiation and IFITM2/3 increasing at later stages (32). Co-expression network analyses further reveal distinct transcriptional modules associated with inflammatory amplification and immune regulation, both dynamically regulated across disease stages (32). In sepsis-associated ARDS, elevated LCN2 expression in monocytes correlates with neutrophil chemokine production, suggesting a role in amplifying pulmonary inflammation and granulocyte recruitment (39).

Neutrophils also undergo substantial remodeling during sepsis. Multiple immature and proliferative subsets, including IL1R2^+^, PADI4^+^, and MPO^+^ immature neutrophils as well as MKI67^+^ CYP1B1^+^ proliferative progenitors, expand significantly, reflecting activation of emergency granulopoiesis (38). Functionally, these neutrophils exhibit immunosuppressive properties, including inhibition of CD4^+^ T-cell proliferation and cytokine production, although this suppressive capacity partially diminishes during recovery (38). Transcriptomic analyses further demonstrate acquisition of myeloid-derived suppressor cell (MDSC)-like features, including enrichment of prostaglandin synthesis and immunosuppressive gene signatures (30). Notably, low-density neutrophils display monocyte-like transcriptional signatures early in disease progression, suggesting that myeloid reprogramming is initiated prior to overt clinical sepsis (32).

MDSCs expand markedly during sepsis, particularly in patients progressing to chronic critical illness. A hybrid subset (H-MDSC) with transcriptional features intermediate between PMN-MDSCs and M-MDSCs has been identified, predominantly in late-stage disease (30). Unlike tumor-associated MDSCs, sepsis-associated MDSCs do not consistently express classical suppressive markers such as ARG1 or IL10, indicating disease-specific regulatory mechanisms (30). In persistent inflammation-immunosuppression-catabolism syndrome (PICS), monocytic populations exhibit an HLA-DR low phenotype associated with immunosuppressive function and poor prognosis, underscoring their role in sustained immune dysfunction (31).

In addition to classical myeloid populations, platelets and megakaryocytes are increasingly recognized as active participants in immune regulation during sepsis. Platelets are elevated in non-survivors and display transcriptional programs related to coagulation, activation, and inflammation (32). Even at early high-risk stages, they exhibit marked transcriptional perturbations and share core inflammatory signatures with monocytes, including S100A8/A9 and interferon-related genes (32), suggesting coordinated regulation of immune and coagulation pathways. Megakaryocytes are also expanded in chronic inflammatory states such as PICS, where specific subsets exhibit immunoregulatory gene signatures and potential anti-inflammatory functions, indicating roles beyond platelet production (31).

### 3.3 T cells and NK cells

Glial cell alterations in sepsis-associated encephalopathy reflect a central component of systemic immune-brain communication rather than an isolated CNS response. In sepsis, CD4^+^ T cells display subset-specific exhaustion and metabolic remodeling. Single-cell analyses reveal enrichment of NR4A2^+^ central memory CD4^+^ T cells in patients with abdominal, pulmonary, and skin-derived sepsis. These cells exhibit an exhausted phenotype characterized by downregulation of CD28 and ICOS and upregulation of PD-L1 and TIM-3, and their high expression levels are associated with poor prognosis (33).

In critical illness, including sepsis, regulatory T cells (Tregs) demonstrate the strongest metabolic plasticity, with enhanced glycolytic capacity that supports maintenance of their frequency and immunosuppressive functions (34). In late-stage sepsis, CD4^+^ T cells display classical “T-cell exhaustion,” with downregulation of ribosomal and mitochondrial genes and upregulation of apoptosis-related genes (27, 29). Similarly, in elderly ARDS patients, several CD4^+^ T-cell-related genes (CD3E, IL7R, CD5, CD247, CD2, CD40LG, ITK, and KLRB1) are markedly downregulated (39). In PICS patients, the proportion of Tregs decreases and shows an inhibitory state characterized by upregulation of DUSP1 and FOS and downregulation of immune regulatory genes, particularly in non-survivors (31). CD8^+^ T cells, NK cells, and NKT cells also exhibit subset expansion and functional imbalance. In adult abdominal and pulmonary sepsis, pro-inflammatory CD8^+^ T, NK, and NKT subsets show upregulated expression of CCL3, CCL4, and TNF, which correlates with high mortality (28). In bacterial sepsis, CD8^+^ T cells show increased expression of cytotoxic genes (GZMA, GZMH, and NKG7) (27). *In vitro* experiments demonstrate that MDSCs suppress CD8^+^ T-cell proliferation and cytokine production, including IFN-γ and IL-2, confirming their immunosuppressive function (30). In the PICS death group, the proportion of CD8 TEMRA cells increases, showing both proliferative activity and immune dysfunction along with upregulation of apoptosis-related genes (31). In elderly ARDS patients, CD8^+^ T cells exhibit increased CD2 expression but decreased CD40LG expression (39). In late-stage sepsis, Th17 cells are largely absent, as reported in multiple studies, suggesting that impaired Th17 differentiation may contribute to immunosuppression and increased susceptibility to secondary infections (27, 29).

### 3.4 B cells and plasma cells

In sepsis, the proportion of B cells generally decreases, and their transcriptomic profiles exhibit metabolic dysfunction and impaired immune regulatory capacity (27). In contrast, plasma cells tend to expand during disease progression, possibly influenced by interferon (IFN) signaling from NK cells (29). Single-cell analyses show marked expansion of plasma cells and plasmablasts in sepsis originating from abdominal, respiratory, and skin infections, and their elevated levels are associated with poor clinical outcomes (33). In PICS patients, the proportions of naïve and memory B cells decline, whereas plasma cells increase but display impaired function. Notably, in PICS survivors, memory B cells and IGHA1^+^ plasma cells exhibit higher activity, suggesting a potential association with improved prognosis (31).

### 3.5 Glial cells in the central nervous system

In sepsis-associated encephalopathy (SAE), glial cells in the hippocampus exhibit pronounced subset-specific functional dysregulation, serving as a critical interface between systemic immune dysregulation and central nervous system inflammation. Among microglia, two distinct subsets can be identified. Microglia 2 display a clear M1-like pro-inflammatory phenotype, characterized by high expression of inflammatory genes such as HLA-DRA, IL1B, and TNF, whereas Microglia 1 show a hypo-responsive state with widespread downregulation of both M1 and M2 marker genes, suggesting functional impairment (35). These transcriptional alterations are closely associated with peripheral inflammatory signaling and systemic immune activation, indicating that circulating cytokines and immune mediators may directly shape microglial functional states during sepsis.

Astrocytes also exhibit marked heterogeneity, with two major subsets identified. Astrocyte 2 simultaneously upregulates both neurotoxic A1 and neuroprotective A2 gene signatures, suggesting a mixed and dysregulated activation state, whereas Astrocyte 1 remains relatively quiescent with reduced A1-associated activity. Notably, both astrocyte subsets show downregulation of genes related to blood-brain barrier (BBB) integrity, providing a molecular basis for BBB disruption during sepsis (35). Such astrocytic dysfunction may further exacerbate systemic immune imbalance by impairing neurovascular integrity and facilitating bidirectional neuro-immune communication, thereby linking central nervous system injury with peripheral immune dysregulation in sepsis.

Beyond classical immune cells, non-immune cells, particularly glial cells, play a central role in shaping neuro-immune interactions during sepsis and should be integrated into the broader framework of systemic immune dysregulation. Microglia and astrocytes respond rapidly to systemic inflammatory cues, including circulating cytokines and pathogen-associated molecular patterns, thereby activating neuroinflammatory pathways.

Importantly, glial activation is tightly coupled with peripheral immune responses. Circulating inflammatory mediators and activated myeloid cells can compromise BBB integrity, enabling peripheral immune signals to access the central nervous system. In turn, activated glial cells release cytokines and chemokines that further amplify systemic inflammation, establishing a bidirectional feedback loop between central and peripheral immune compartments.

Single-cell and spatial transcriptomic studies further reveal substantial heterogeneity in glial states during sepsis, including pro-inflammatory and neuroprotective subpopulations. These distinct states are associated with differential regulation of metabolic programs, phagocytic activity, and neuronal support functions. For instance, astrocytes exhibit context-dependent roles in maintaining BBB integrity, regulating neurotransmitter homeostasis, and modulating immune signaling.

Furthermore, emerging evidence suggests that systemic immune dysregulation, particularly myeloid cell reprogramming and persistent inflammatory signaling, contributes to long-term neurocognitive impairment in sepsis survivors. Therefore, integrating glial cell responses with systemic immune dynamics provides a more comprehensive framework for understanding SAE, linking peripheral immune alterations to central nervous system dysfunction and highlighting potential therapeutic targets for intervention.

## 4 Key mechanisms of immune regulation in sepsis

Immune regulation in sepsis is characterized by a multilevel and multi-mechanistic dynamic imbalance. Single-cell transcriptomic studies have revealed that immunosuppression and inflammation coexist throughout sepsis progression. T-cell exhaustion, co-expression of pro-inflammatory and inhibitory genes, abnormal expansion of neutrophils, and transcriptional reprogramming of platelets and hematopoietic progenitors collectively contribute to a state often described as immune paralysis. Meanwhile, immune cells undergo metabolic reprogramming, shifting from oxidative phosphorylation to glycolysis, while regulatory T cells (Tregs) and monocytes enhance survival and suppressive functions through the kynurenine metabolic pathway.

**Figure 3:**
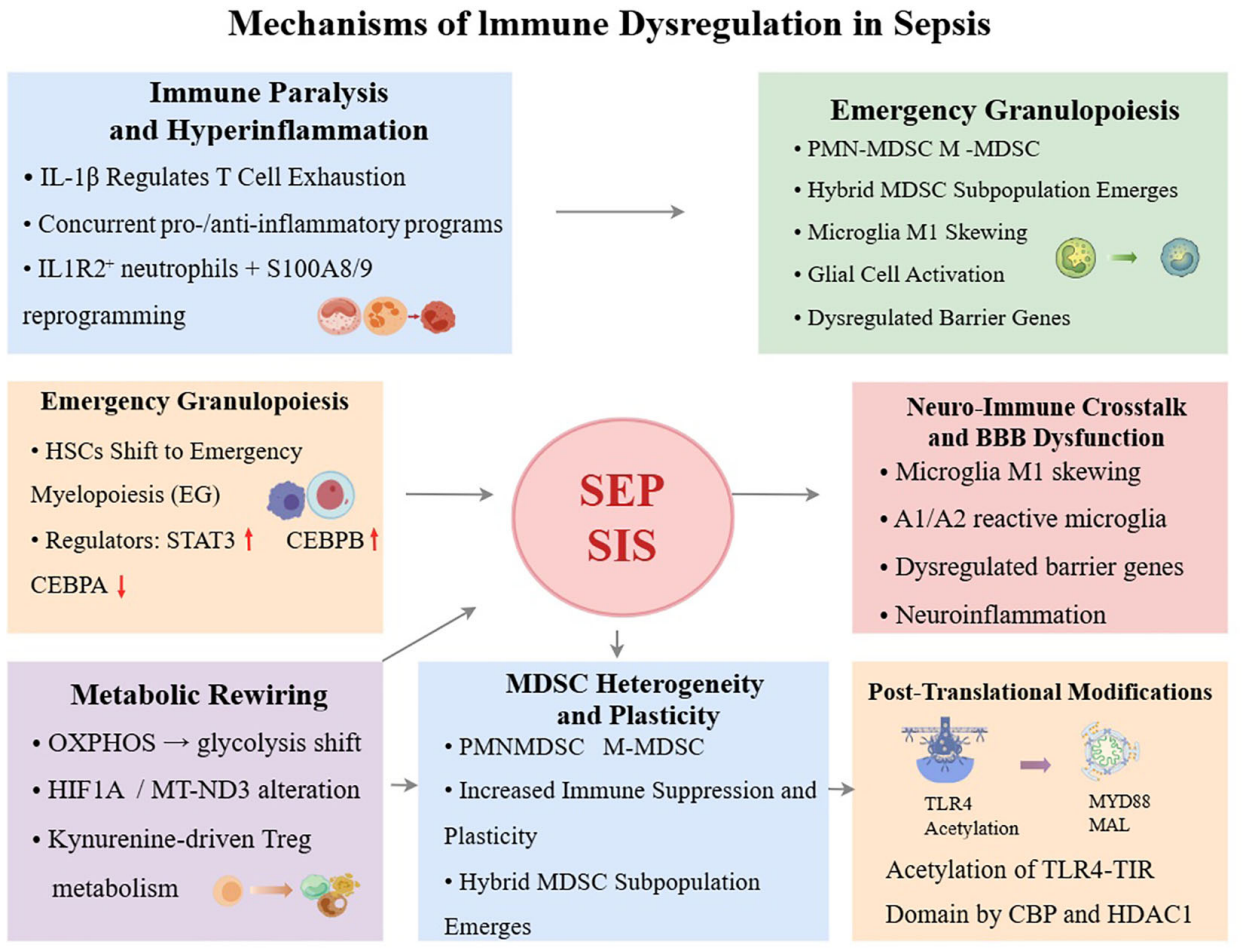
Analysis diagram of the immune regulation mechanism in sepsis, covering key regulatory information such as MDSC heterogeneity, SRS1, HSC emergency granulopoiesis, Treg/KYN metabolism, coexistence of inflammation and immune suppression, neuro-immune interaction, and PTM modification.

Emergency granulopoiesis (EG) is activated in circulating hematopoietic stem cells, where CEBPB and STAT3 drive EG, whereas CEBPA maintains steady-state granulopoiesis, providing the transcriptional basis for neutrophil dysregulation. Myeloid-derived suppressor cells (MDSCs) exhibit marked heterogeneity and plasticity, dynamically transitioning between inflammatory and immunosuppressive states. Sepsis also induces neuro-immune interactions, where blood–brain barrier disruption and aberrant communication among glial and endothelial cells exacerbate neuroinflammation. Furthermore, post-translational modifications, such as TLR4 acetylation and haptoglobin glycosylation, fine-tune immune signaling intensity. Infection sources, age groups, and clinical contexts further shape immune responses, leading to highly individualized immune regulation patterns. Collectively, these mechanisms form a systems-level framework for understanding immune dysregulation in sepsis, providing a theoretical basis for precision diagnosis and treatment (**Figure 3**).

### 4.1 Coexistence of immunosuppression and inflammation

In late-stage sepsis, the immune landscape is characterized by the simultaneous presence of pro-inflammatory responses and immunosuppression, with immune cells co-expressing inflammatory and inhibitory genes, resulting in a state termed immune paralysis (27, 29). Monocytes regulate T-cell activation and exhaustion through IL-1β signaling, further exacerbating immune imbalance (29).

Expansion and persistence of myeloid-derived suppressor cells (MDSCs) represent a hallmark of post-sepsis immunosuppression and are closely associated with the development of chronic critical illness (CCI) (30). In CD4^+^ T cells, NR4A2 drives T-cell exhaustion; its deletion improves survival in mouse models, whereas overexpression worsens disease progression, highlighting its key regulatory role in T-cell dysfunction during sepsis (33). Regulatory T cells maintain suppressive activity under mitochondrial stress by acquiring enhanced glycolytic capacity, while maintaining stable expression of FOXP3 and TIGIT, whereas conventional T cells exhibit impaired IL-2 production, underscoring the importance of metabolic adaptation in immune imbalance (34). Post-translational modifications and receptor-mediated signaling also play crucial roles in immune regulation. Fucosylated haptoglobin (Fu-Hp) activates macrophage-like cells through the Mincle receptor, enhancing expression of inflammatory cytokines such as IL-1β, IL-6, and TNF and triggering activation of the NLRP3 inflammasome. Notably, this pathway is weakened in non-survivors, suggesting its association with dysregulated immune responses (37). Additionally, reversible acetylation of the TLR4-TIR domain, mediated by CBP and reversed by HDAC1, functions as a molecular switch that modulates activation of the TLR4/MAL/MyD88 signaling pathway, thereby influencing M1 macrophage polarization and systemic inflammation (36).

In patients with persistent inflammation, immunosuppression, and catabolism syndrome (PICS), immune function remains severely impaired, characterized by suppressed monocyte function, exhaustion of B and T cells, and enhanced immunomodulatory activity of megakaryocytes. Cell communication analysis reveals that intercellular signaling strength and pathway activity are markedly reduced in PICS non-survivors, reflecting global immune system failure (31). Clinical contexts further highlight the complexity of immunosuppression. In COVID-19-associated sepsis and elderly ARDS patients, hub genes in T cells (e.g., CD247, CD2, CD40LG) are downregulated and negatively correlated with key inflammatory mediators (IFN-γ, IL-6) and chemokines (KC, CCL-2), suggesting that their suppression may aggravate inflammatory–immunosuppressive imbalance (39). A neutrophil-driven immunosuppressive subtype (SRS1) is characterized by expansion of IL1R2^+^ immature neutrophils and proliferating neutrophil precursors, accompanied by monocytopenia. Patients with this subtype show stronger neutrophil-mediated suppression of CD4^+^ T cells, impaired phagocytosis, and higher early mortality, indicating a distinct disease endotype driven by dysregulation of the neutrophil–emergency granulopoiesis axis (38).

In neonatal sepsis, particularly in preterm infants, significant lymphopenia, reduced dendritic cell frequency, and decreased monocyte HLA-DR expression can distinguish infection even in the absence of elevated CRP. Additionally, AREG emerges as a key component of early host responses and may exert protective effects through tissue repair mechanisms (40). Early transcriptional reprogramming of myeloid cells, including monocytes and platelets, represents a critical event during the transition from high-risk states to clinical sepsis. Persistent upregulation of genes such as S100A8/A9 and IFITM2/3 may serve as a bridge between early inflammatory activation and subsequent immune dysregulation (32).

Overall, late-stage immune imbalance in sepsis exhibits a highly complex network structure, driven by concurrent inflammation and immunosuppression, metabolic adaptation, cellular plasticity, and transcriptional reprogramming.

### 4.2 Metabolic reprogramming

Metabolic reprogramming is a central mechanism underlying immune dysregulation in sepsis, not only reflecting altered energy utilization but also directly shaping immune-cell effector functions. Single-cell transcriptomic analyses reveal that multiple immune cell types undergo a metabolic shift from oxidative phosphorylation (OXPHOS) to glycolysis, particularly in monocytes and platelets (28). This transition is mechanistically linked to rapid inflammatory activation, including increased cytokine production and platelet activation, while simultaneously predisposing cells to dysfunctional or exhausted states under sustained stress. Activation of hypoxia-inducible signaling pathways is a key driver of this process. In particular, upregulation of HIF1A is associated with the expansion of erythroid progenitors and correlates with poor prognosis and increased mortality risk, suggesting that hypoxia-driven metabolic adaptation contributes to pathological hematopoietic reprogramming in sepsis.

At the cellular level, metabolic states are tightly coupled to immune-cell functional phenotypes. In early sepsis, monocytes and macrophages exhibit a glycolytic switch driven in part by HIF-1α signaling, which supports rapid ATP generation and promotes pro-inflammatory cytokine release. This metabolic transition is often accompanied by increased pentose phosphate pathway (PPP) activity, providing NADPH for reactive oxygen species (ROS) production and antimicrobial responses. As sepsis progresses, however, sustained metabolic stress induces a shift toward immunosuppressive states. Mitochondrial dysfunction, reduced OXPHOS capacity, and accumulation of metabolic intermediates such as lactate contribute to impaired antigen presentation and decreased HLA-DR expression in monocytes.

Within myeloid-derived suppressor cell (MDSC) populations, metabolic heterogeneity further supports functional specialization. H-MDSCs exhibit high expression of mitochondrial genes, including MT-ND1 and MT-ATP6, indicating a reliance on OXPHOS that may sustain their survival and reinforce immunosuppressive capacity (30), thereby promoting T-cell dysfunction and impaired pathogen clearance. In neutrophils, metabolic rewiring is closely associated with emergency granulopoiesis and functional plasticity. Immature subsets rely predominantly on glycolysis, whereas MDSC-like neutrophils show enrichment of prostaglandin synthesis and arginine metabolism pathways, enhancing their ability to suppress T-cell activation and contributing to immune paralysis (38).

Adaptive immune cells also display pronounced metabolic heterogeneity. Among CD4^+^ T-cell subsets, regulatory T cells (Tregs) acquire enhanced glycolytic capacity under critical illness, enabling persistence in nutrient-deprived environments and maintaining suppressive function partly through the kynurenine metabolic pathway (34). In addition, Tregs may engage fatty acid oxidation and oxidative metabolism to further support their stability and immunosuppressive activity under inflammatory conditions. These adaptations collectively contribute to sustained immunosuppression in late-stage sepsis.

At the systems level, transcriptional regulatory networks further integrate metabolic and immune programs. Co-regulation analysis reveals a shared dysregulated network between monocytes and platelets during sepsis progression, characterized by upregulation of SPI1, JDP2, and STAT2 and downregulation of STAT6 (32). Among these factors, SPI1 (PU.1) is predicted to function as a central upstream regulator that coordinates metabolic and inflammatory gene expression programs, including S100A8, thereby linking metabolic reprogramming to immune activation across multiple cell types.

Importantly, metabolic regulation is closely intertwined with post-translational modifications. For example, acetyl-CoA availability influences histone acetylation and TLR4 signaling, while metabolite-dependent modifications such as lactylation may regulate gene expression programs associated with immune tolerance. Collectively, these findings demonstrate that metabolic reprogramming in sepsis is not a passive consequence of cellular stress but a key driver of immune-cell functional specialization, directly orchestrating inflammatory activation, immunosuppression, and long-term immune exhaustion.

### 4.3 Emergency granulopoiesis

Sepsis, particularly the SRS1 endotype, is strongly associated with enhanced emergency granulopoiesis (EG) in circulating hematopoietic stem cells (HSCs) (38). The transcription factor CEBPB acts as a key regulator driving EG, whereas CEBPA primarily maintains steady-state granulopoiesis (SSG) (38). STAT3 plays a central driving role in SRS1 patients. Its associated gene expression programs are upregulated across multiple neutrophil subsets, accompanied by increased plasma levels of cytokines such as G-CSF and IL-6, which further reinforce activation of the EG axis through STAT3 signaling (38). Single-cell analyses further reveal cluster-specific regulatory mechanisms within HSC populations. The C5 HSC cluster, driven by the CEBPB–STAT3 axis, represents an EG-associated cluster significantly enriched in SRS1 patients, whereas cluster C7 is more closely associated with CEBPA-dominated SSG programs. Computational knockout and overexpression simulations confirm the essential roles of CEBPA, CEBPB, and STAT3 in maintaining the identities of the C7 (SSG) and C5 (EG) clusters (38). These findings define the transcriptional regulatory basis of granulopoiesis imbalance in sepsis and provide a mechanistic framework for understanding abnormal neutrophil expansion and its immunosuppressive functions.

### 4.4 Heterogeneity and dynamic plasticity of MDSCs

Single-cell transcriptomic studies demonstrate that myeloid-derived suppressor cells (MDSCs) in sepsis do not represent a single terminally differentiated population but instead exhibit pronounced lineage continuity and functional plasticity (30). High-resolution transcriptional mapping combined with RNA velocity analysis reveals dynamic transitions among different MDSC subsets. Monocytic MDSCs (M-MDSCs) may represent transitional states between early suppressive progenitors and mature myeloid cells such as monocytes and dendritic cells, suggesting a bridging role between inflammation and immunosuppression. In contrast, H-MDSCs exhibit higher state plasticity, potentially undergoing functional reprogramming under different inflammatory microenvironments (30). These findings challenge the traditional view of MDSCs as a fixed terminal suppressive population.

Single-cell phenotypic and functional heterogeneity also suggests that classical surface markers used in tumor studies may not fully apply to sepsis. For instance, CD15, commonly used to identify PMN-MDSCs, shows limited discriminative power in sepsis, whereas CD66b may provide improved identification but still exhibits substantial transcriptional and functional heterogeneity (30). Therefore, multidimensional definitions integrating transcriptional signatures, trajectory analysis, and functional pathway enrichment are likely required to accurately characterize the biological roles of MDSCs in sepsis-associated immune dysregulation.

### 4.5 Neuroimmune interactions and blood–brain barrier dysfunction

Sepsis affects not only the peripheral immune system but also reshapes the central nervous system through neuroimmune interactions. Single-nucleus transcriptomic analyses reveal extensive remodeling of the neurovascular unit in the hippocampus, involving microglia, astrocytes, endothelial cells, and pericytes (35). Microglia display a shift toward M1-like pro-inflammatory phenotypes, while astrocytes exhibit mixed A1/A2 activation states, jointly driving neuroinflammatory processes (35). Cell communication analysis using CellChat indicates enhanced inflammatory signaling pathways—including TGF-β, PDGF, and PECAM1—between microglia, astrocytes, and vascular cells (35). Simultaneously, blood–brain barrier (BBB)-related gene expression becomes dysregulated, with downregulation of barrier-supporting pathways such as angiogenesis and Notch signaling and upregulation of permeability-related genes. These changes provide a molecular basis for sepsis-associated encephalopathy (SAE) (35). These findings highlight neuroimmune dysregulation as an important component of multi-organ dysfunction in sepsis.

### 4.6 Role of post-translational modifications

Beyond transcriptional regulation and metabolic reprogramming, post-translational modifications (PTMs) serve as key molecular mechanisms for fine-tuning immune signaling intensity in sepsis. Recent molecular and single-cell studies demonstrate that acetylation and glycosylation modifications reshape inflammatory signaling pathways by altering receptor conformation and ligand interactions. In terms of acetylation regulation, CBP-mediated acetylation of the TLR4-TIR domain functions as a molecular switch controlling activity of the TLR4/MAL/MyD88 signaling pathway, while HDAC1 mediates its deacetylation, forming a reversible regulatory mechanism (36). This acetylation modification is significantly increased in CD16^+^ monocytes of sepsis patients and positively correlates with systemic inflammation levels, suggesting that it enhances TLR4 signaling, promotes M1 macrophage polarization, and amplifies inflammatory responses (36). At the glycosylation level, terminal fucosylation of haptoglobin (Hp) is significantly increased in sepsis, particularly at Asn207 and Asn211 sites (37). This abnormal glycoform, fucosylated Hp (Fu-Hp), activates macrophage-like cells through the C-type lectin receptor Mincle (CLEC4E), enhancing production of IL-1β, IL-6, and TNF and inducing activation of the NLRP3 inflammasome, thereby intensifying inflammatory cascades (37). Further studies indicate that FUT4, a fucosyltransferase, is significantly upregulated in monocytes/macrophages of sepsis patients and positively correlates with Hp fucosylation levels, suggesting that FUT4 may act as a key enzymatic regulator driving abnormal Hp glycosylation (37). Collectively, TLR4 acetylation and the Fu-Hp/Mincle glycosylation signaling axis amplify inflammatory responses through complementary mechanisms, providing a molecular basis for persistent inflammatory activation in sepsis. Targeting these pathways may represent novel therapeutic strategies for restoring immune homeostasis.

### 4.7 Infection source- and age-specific immune responses

Different anatomical infection sources shape distinct immune transcriptional programs, representing an important source of immune heterogeneity in sepsis. Single-cell transcriptomic analyses show that infections originating from the abdomen, lungs, urinary tract, skin, or central nervous system are associated with distinct distributions and functional states of monocyte and lymphocyte subsets. For example, urosepsis patients exhibit specific monocyte subset distributions, which may be associated with relatively favorable clinical outcomes (33). These findings suggest that infection sites influence not only inflammatory intensity but also disease progression by modulating myeloid differentiation trajectories and immune regulatory capacity. At a broader level, certain immune endotypes are conserved across infection contexts. The SRS1 endotype, characterized by expansion of IL1R2^+^ immature neutrophils and STAT3-driven gene expression programs, has been validated across multiple pathogens (e.g., SARS-CoV-2 and influenza virus), infection sources (community-acquired pneumonia and fecal peritonitis), clinical syndromes (ARDS), and age groups (pediatric septic shock) (38). These findings suggest that SRS1 may represent a conserved extreme host response endotype centered on dysregulated emergency granulopoiesis. Age further modulates immune responses. While pediatric and adult sepsis share certain inflammatory signatures, they also display notable differences, such as child-specific monocyte subset distributions (33). In elderly populations, T-cell hub gene expression patterns in sepsis-induced ARDS significantly overlap with those observed in COVID-19 and sepsis, suggesting increased immune vulnerability and inflammatory amplification under co-infection conditions (39). In extremely preterm infants, sepsis exhibits distinct immune characteristics, including pronounced lymphopenia, reduced dendritic cell frequency, and early upregulation of AREG (40). These features likely reflect the rapid developmental stage of the neonatal immune system and its high susceptibility to infection. Pathogen type also significantly influences adaptive immune programs. For example, bacterial and fungal sepsis differ in CD8^+^ T-cell cytotoxic gene expression profiles, indicating that different microbial components may shape T-cell functional states through distinct antigenic stimuli and innate immune signaling pathways (27).

Immune regulation in sepsis involves both conserved endotype mechanisms across infections and context-specific transcriptional programs shaped by infection site, pathogen type, and host age. This dual framework of shared mechanisms and contextual modulation provides an important theoretical basis for understanding immune heterogeneity and developing precision immunophenotyping strategies in sepsis.

## 5 Potential biomarkers and therapeutic targets

**Figure 4:**
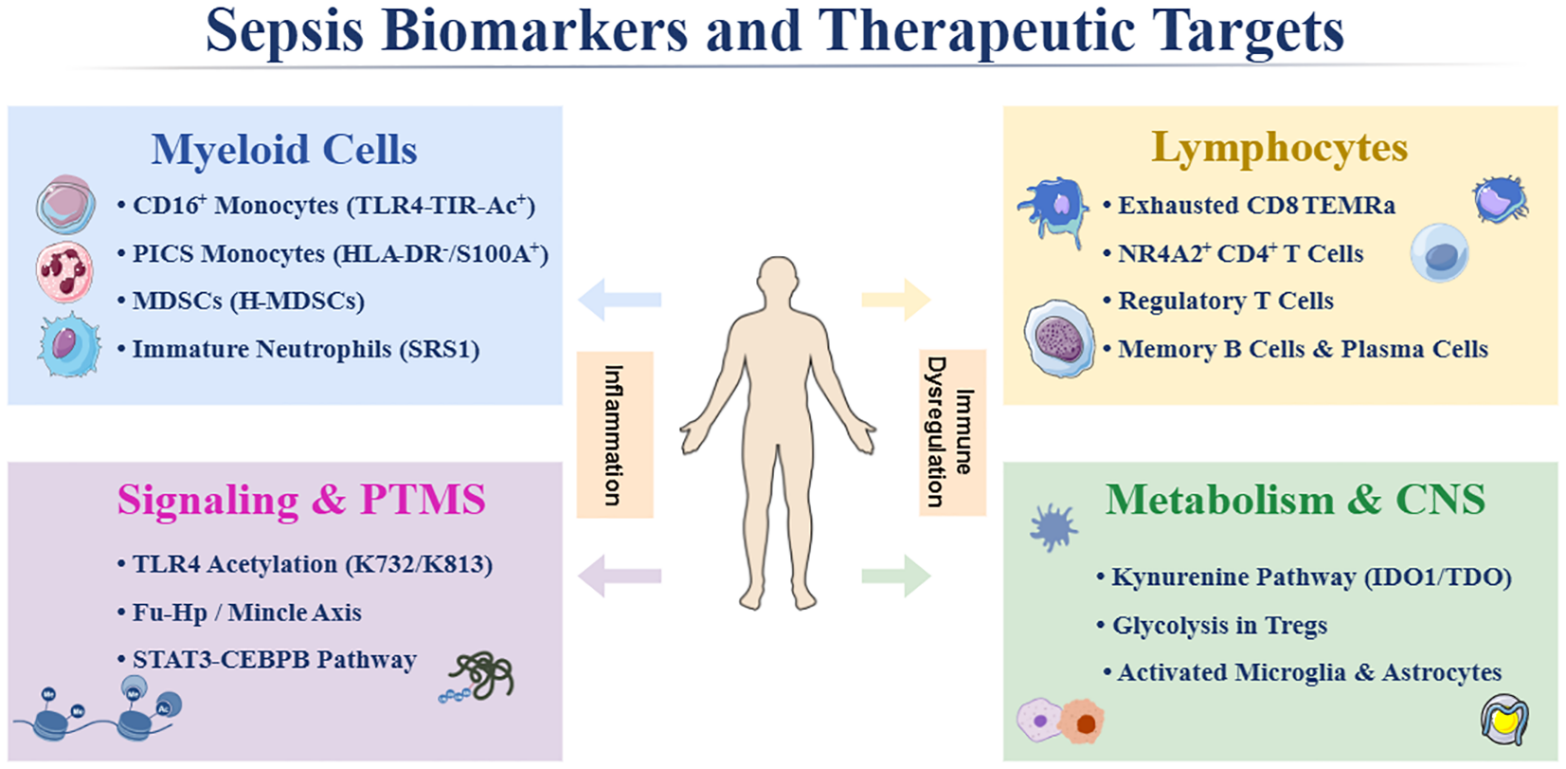
Framework diagram of sepsis biomarkers and therapeutic targets, covering various biomarker information related to myeloid cells, lymphocytes, glial cells, signaling pathways, and metabolism.

Sepsis is a complex syndrome characterized by the coexistence of systemic inflammation and profound immune dysregulation, with highly heterogeneous clinical manifestations and rapid disease progression. In recent years, multi-omics and single-cell studies have identified a variety of potential molecular and cellular biomarkers. These markers not only reflect the inflammatory status and degree of immune suppression in patients with sepsis but also provide important clues for early diagnosis, prognostic assessment, and precision intervention. They span multiple biological levels, including myeloid cells, lymphocytes, platelets, hematopoietic progenitor cells, and central nervous system cells, and involve regulatory mechanisms such as transcriptional control, metabolic reprogramming, and post-translational modifications. Systematic integration of these biomarkers will help construct a comprehensive immune–metabolic regulatory map of sepsis and provide a theoretical basis for future immunomodulatory and multi-target therapeutic strategies (**Figure 4**). .

### 5.1 Post-translational modifications and signaling pathways

During sepsis, dynamic regulation of post-translational modifications (PTMs) and signaling pathways plays a central role in modulating myeloid cell function and systemic inflammatory responses. Acetylation of the TLR4-TIR domain (K732/K813) represents a key PTM that regulates TLR4 signaling activity and promotes M1 macrophage polarization, thereby amplifying inflammatory responses. The level of this modification is closely associated with inflammatory activity in sepsis and may serve as a potential molecular biomarker. Pharmacological agents such as CBP inhibitors or HDAC1 agonists may restore immune balance by modulating this modification (36). In addition, the Fu-Hp/Mincle signaling axis has been identified as an important pathway driving macrophage inflammatory responses and activation of the NLRP3 inflammasome in sepsis. Activation of this pathway not only reflects disease inflammatory activity but also provides opportunities for targeted intervention. Inhibiting the interaction between Fu-Hp and Mincle may reduce inflammation-associated tissue damage (37). Regarding the regulation of emergency granulopoiesis (EG), the STAT3–CEBPB axis drives the expansion of immature neutrophils and contributes to the formation of the SRS1 endotype, representing an important mechanism of myeloid reprogramming in sepsis. Targeting STAT3, CEBPB, or their upstream cytokine pathways (e.g., G-CSF and IL-6) may provide potential therapeutic strategies to correct early myeloid immune dysregulation by modulating abnormal granulopoiesis (38).

### 5.2 Myeloid cell biomarkers

Myeloid cells are key effector populations in both inflammation and immunosuppression during sepsis. Highly inflammatory CD16^+^ monocytes (characterized by high levels of TLR4-TIR acetylation) correlate with sepsis severity and may serve as biomarkers of inflammatory activity (36). Monocyte subsets associated with persistent inflammation, immunosuppression, and catabolism syndrome (PICS), such as Mono1/Mono4, exhibit an immunosuppressive phenotype characterized by HLA-DR^low and functional impairment, resembling monocytic myeloid-derived suppressor cells (M-MDSCs). These cells may serve as biomarkers for the diagnosis and prognostic evaluation of PICS (31). Moreover, myeloid-derived suppressor cells (MDSCs)—particularly the H-MDSC subset—expand persistently in patients with chronic critical illness, suggesting their role in maintaining long-term immunosuppression (30). Immature neutrophils and the SRS1 transcriptional signature can function as early transcriptomic biomarkers predicting immunosuppression and high-risk patient groups. In addition, the STAT3–CEBPB–regulated emergency granulopoiesis pathway provides a potential target for early intervention in abnormal myeloid activation during sepsis (38).

### 5.3 Lymphocyte biomarkers

Functional alterations in lymphocyte subsets also provide important biomarkers in sepsis and PICS. In PICS patients, CD8 TEMRA cells and regulatory T cells (Tregs) exhibit an imbalance between proliferation and exhaustion, reflecting suppressed cellular immunity and increased mortality risk (31). High expression of NR4A2 in CD4^+^ T cells drives T-cell exhaustion and may serve both as a prognostic biomarker and a potential target for immunotherapy, such as NR4A2 inhibitors (28). Expression levels of CD52 in lymphocytes correlate with cellular activation status and may serve as predictors of patient survival or targets for immune-enhancing therapies (33). In addition, pro-inflammatory mediators such as CCL3, CCL4, and TNF are upregulated in CD8^+^ T cells, NK cells, and NKT cells, reflecting lethal inflammatory states (33). Changes in B-cell and plasma-cell subsets also reflect immune recovery potential. Active memory B cells and IGHA1^+^ plasma cells show higher activity in the PICS survival group and may serve as positive indicators of immune restoration (31). Furthermore, the glycolytic capacity of Tregs and the kynurenine metabolic pathway (IDO1/TDO/KMO) reflect immunosuppressive and metabolic adaptation states, providing potential targets for pleiotropic therapeutic strategies (34).

### 5.4 Glial cells and other potential targets

The polarization state of glial cells in the central nervous system plays a crucial role in sepsis-associated encephalopathy (SAE). The M1-like polarization of microglia and the A1/A2 activation states of astrocytes are closely associated with neuroinflammation and disruption of the blood–brain barrier (BBB), providing potential therapeutic strategies targeting glia–vascular communication (35). Early activation signatures of platelets and megakaryocytes reflect coagulation–immune interactions during the early stages of sepsis. The transcription factor SPI1 (PU.1) coordinates inflammatory gene programs in both monocytes and platelets and may serve as a potential target for intervention in early myeloid dysregulation (32). In addition, inflammatory mediators shared across different age groups and infection sources, such as IL-6 and EN-RAGE, as well as features observed in preterm neonatal sepsis, including lymphopenia, reduced dendritic cell frequency, and decreased HLA-DR expression, may serve as biomarkers for early diagnosis and immune status assessment (28, 38).

## 6 Conclusion and perspectives

In recent years, advances in single-cell transcriptomics, epigenomics, metabolic profiling, and integrative multi-omics analyses have profoundly expanded our understanding of the complexity and dynamic nature of the immune system in sepsis. These studies demonstrate that sepsis not only reshapes the composition of peripheral immune cells but also influences the developmental trajectories of central nervous system glial cells, hematopoietic stem/progenitor cells (HSCs/HSPCs), and bone marrow myeloid lineages. Immune cell functions vary markedly across disease stages, infection sources, age groups-including preterm neonates, children, adults, and the elderly-and tissue microenvironments. For example, myeloid cells undergo dynamic reprogramming from highly inflammatory monocyte/macrophage states to immunosuppressive M-MDSC-like phenotypes; neutrophils exhibit immature features driven by emergency granulopoiesis; T cells display exhaustion and metabolic adaptation; and B cells show functional imbalance in persistent inflammation, immunosuppression, and catabolism syndrome (PICS). In parallel, glial cells demonstrate impaired blood dependent inflammatory or protective phenotypes, while AREG-mediated immunoregulation and tissue repair signaling are prominently activated in preterm neonatal sepsis. These findings collectively highlight multi-layered immune regulation across systemic and central compartments. Moreover, immune dysregulation is closely linked to metabolic reprogramming and post-translational modifications, including TLR4-TIR acetylation, the Fu-Hp/Mincle signaling axis, and STAT3-CEBPB-driven emergency granulopoiesis, providing an integrated framework for understanding sepsis immunopathology.

Despite these advances, several critical challenges remain. Current studies are still largely focused on patients after clinical diagnosis, whereas early immune alterations in high-risk populations remain insufficiently characterized. Establishing longitudinal cohorts with multi-timepoint sampling and integrating single-cell sequencing with metabolic profiling may enable identification of early immune imbalance signatures, such as monocyte-associated gene patterns and platelet activation signals, which could be combined with clinical indicators to support early warning and intervention strategies. In addition, the relatively small sample sizes and limited cohort diversity in many studies constrain the ability to capture the full heterogeneity of sepsis. Future research should therefore incorporate larger, multicenter cohorts spanning different clinical outcomes, age groups, and complications, enabling robust validation of newly identified immune subsets and improving generalizability.

Another major challenge lies in resolving the dynamic evolution of immune dysregulation across disease stages. Integrative multi-omics approaches that combine scRNA-seq, scATAC-seq, single-cell metabolic assays, and post-translational modification profiling across multiple time points will be essential for constructing comprehensive immune-metabolic-modification landscapes. Such analyses may clarify mechanisms underlying abnormal myelopoiesis, inflammatory amplification, and progressive immunosuppression. At the same time, incorporation of spatial multi-omics and *in situ* tissue analyses will provide critical insights into how tissue microenvironments shape immune cell function, particularly in organs such as the brain and bone marrow, thereby improving our understanding of organ-specific pathology, tissue repair, and chronic critical illness.

Importantly, although single-cell studies have identified numerous candidate molecular targets and immune cell subsets, functional validation remains limited. Future work should integrate experimental approaches, including *in vitro* co-culture systems, gene-edited animal models, and CRISPR-based perturbation strategies, to elucidate the mechanistic roles of key regulators and assess their therapeutic potential. Building on these insights, the development of early intervention strategies targeting key immune pathways, such as modulation of inflammatory amplification, correction of abnormal myelopoiesis, and restoration of immune balance, represents a promising direction. Ultimately, the integration of multi-omics data with clinical parameters is expected to facilitate the development of predictive models for early diagnosis, risk stratification, and personalized therapy.

In summary, multi-omics studies have substantially advanced our understanding of systemic and central immune mechanisms in sepsis. Continued integration of high-dimensional data, mechanistic validation, and clinical translation may enable the establishment of a closed-loop framework, from early detection to targeted intervention, thereby improving the precision and effectiveness of sepsis management.
